# Influence of various fabrication techniques and porcelain firing on the accuracy of metal-ceramic crowns

**DOI:** 10.1186/s12903-024-04634-7

**Published:** 2024-07-26

**Authors:** İlknur Usta Kutlu, Yeliz Hayran

**Affiliations:** 1 Department of Prosthodontics, Faculty of Dentistry, Gaziosmanpasa University, Tokat, Turkey; 2 Department of Prosthodontics, Faculty of Dentistry, Uludağ University, Bursa, Turkey

**Keywords:** CAD/CAM, Selective laser sintering, Selective laser melting, Soft metal milling, Hard metal milling, Discrepancy

## Abstract

**Background:**

The fit of a metal-ceramic restoration is essential to its long-term durability. Regarding marginal and internal fit, there is not enough information about the technologies used in the production of metal-ceramic restorations. The aim of this in vitro study is to compare, both before and after porcelain firing, the marginal, axial, axio-occlusal, and occlusal fit of metal-ceramic restorations manufactured using casting, additive or subtractive computer-aided design, and computer-aided manufacturing techniques (CAD/CAM).

**Methods:**

CAD/CAM were used to create 50 prepared maxillary first molar-shaped Co-Cr die models, which were randomly divided into 5 groups (*n* = 10). Cobalt-chrome copings were produced by casting (C), hard metal milling (HM), soft metal milling (SM), selective laser melting (SLM), and selective laser sintering (SLS) techniques. Before and after porcelain firing, discrepancies of the copings were measured using the silicone replica technique. The data obtained by measurements with a stereomicroscope at x80 magnification were analyzed statistically in the SPSS program. The ROBUST three-way analysis of variance (ANOVA) method was used to compare the discrepancy values.

**Results:**

There were statistically significant differences among fabrication methods (*P* < .001). The HM method showed the highest discrepancy (90.1 μm), and the C (63 μm) method showed the lowest discrepancy in terms of the die model- crown fit. The C, SLS, and SM methods (63 μm; 61.6 μm; 67.7 μm) were statistically similar (*P* > .001). The highest discrepancy was observed on the occlusal area (87.1 μm), and the lowest discrepancy was observed on the axial area (47.7 μm) of the coping. Porcelain firing had a decrease in the discrepancy values (*P* = .001).

**Conclusion:**

All CAD/CAM techniques are appropriate for clinical use; selective laser sintering and soft milling can be the more recommended methods for the compatibility of metal-porcelain restorations, as they have lower discrepancy values than the SLM and HM methods.

## Background

Metal porcelain restorations are widely used in clinical settings as a standart treatment, even though their efficacy varies depending on several factors. When considering retention, crown fit plays an essential role in a cast restoration’s clinical acceptability [1–3]. An increase in the marginal gap leads to increased cement dissolution and plaque retention, which can be harmful to the periodontal tissues and teeth [4–6]. For an appropriate luting, the internal gap, which was stated as the vertical distance between the coping and the abutment teeth, should be uniform at the occlusal/ incisal, and axial surfaces [2]. The resistance of the restoration to vertical and horizontal forces is increased by a superior internal fit [7, 8]. According to recent studies, the fabrication method also affects the marginal fit of Co-Cr dental alloys [9–14].

Both noble and non-noble alloys can be used to create metal-ceramic restorations. Cobalt-chromium (Co-Cr) alloys have long been used in the casting process to create metal-ceramic restorations because of their affordability, biocompatibility, high hardness, longevity, and resistance to corrosion [1, 15–17]. The process of conventionally casting the framework for a prosthesis is labor-intensive, time-consuming, and fraught with problems such as distorted wax patterns and irregularities in the cast metal [18–20]. By producing blanks in highly standardized industrial settings, CAD-CAM milling technologies, whether additive or subtractive, eliminate porosity and flaws related to casting [21].

Both fully sintered and presintered alloy blocks can be utilized with the subtractive CAD-CAM technology [13, 22]. Hard alloy blocks that are non-porous can be milled using the hard metal milling (HM) technique to create metal copings. Nevertheless, it is challenging to mill hard-sintered (non-porous) blocks, and milling equipment quickly overheats and wears out. The milled coping, however, does not require another sintering procedure [23–27].

In substractive CAD/CAM technology, the soft metal milling (SM) technique can also be used to produce metal copings. This technique is more feasible because the blocks are pre-sintered. When compared to milling hard metal blocks, soft metal milling produces comparatively less heat, requires no water cooling, and has the benefit of reducing machine stress. This prolongs the life of consumables like rotary cutting tools and cuts down on milling time [2, 25, 26, 28]. To eliminate porous structures and achieve full density, soft milled copings must be sintered in an argon gas atmosphere [24–26].

Another current CAD/CAM technology used in the production of CoCr metal substructures is additive manufacturing, also known as 3D printing or rapid prototyping. The most popular additive manufacturing technology for processing metals in dentistry is powder bed fusion, which includes selective laser sintering (SLS), selective laser melting (SLM), and electron beam melting [22, 29–31]. Using a high-temperature laser to turn CAD information into a three-dimensional structure, CoCr powders are selectively irradiated, and the irradiated portion melts to form a thin layer. Copings with the required shape are created layer by layer, repeating this procedure in a powder bed [23, 24, 26, 32, 33]. The physical and mechanical properties of metal products are directly impacted by the fusion of metal particles with either the SLS or SLM technique. While SLM can produce metals with a full density of about 99.8%, fusing powder particles with SLS results in low-density metals [29, 30, 34]. Alloy particle fusion can be accomplished using a variety of lasers, such as CO_2_ lasers, ytterbium fiber lasers, and Nd-YAG lasers. The energy of the chosen laser must be high enough to fuse the alloy particles. In general, laser energy increases with decreasing wavelength. The alloy powder is entirely melted by SLM using a high-power laser [11, 16, 22, 30]. Although SLM and SLS methods produce less waste than CAD/CAM milling, they also have drawbacks such as interlayer delamination, balling phenomenon, and porosity [7].

Similar to metal production techniques, porcelain firing also has an impact on the marginal fit and, consequently, the success of the prosthetic restoration. The different coefficients of thermal expansion between the metal and the ceramic may result in dimensional distortion due to heat changes during the firing and cooling processes [22, 32].

Despite the potential of these approaches to construct prostheses, additional laboratory and clinical testing is required to ensure that dental restorations made using these procedures are at least as successful as those produced using conventional casting. There is insufficient research analyzing all metal production methods collectively, despite several studies examining how different procedures affect the fit of metal-ceramic restorations in various combinations. While some consider hard milling [1, 4, 27, 31, 35, 36] or soft milling successful in terms of accuracy [9, 12, 18], others find laser sintering [3, 9, 23, 32] or laser melting [4, 23] effective. Studies on the effect of porcelain firing are limited. There have been reports suggesting that porcelain firing increases the discrepancy [23, 32]. However, some studies indicate that repeated firings do not necessarily lead to a significant increase in the discrepancy [13, 16, 18, 31] and may even improve the fit. [11, 37] Therefore, the purpose of this study was to assess, both before and after porcelain firing, the accuracy of metal-porcelain crowns made by hard metal milling, soft metal milling, selective laser sintering, selective laser melting, and conventional casting techniques. The null hypothesis was that neither the metal fabrication techniques nor the porcelain firing or measurement area would affect the accuracy of the metal-porcelain restorations.

## Methods

Sample size was determined using a power analysis conducted with G Power 3.1.9.4 software (α = 0.05, effect size = 0.45, 80% power). The calculation indicated a minimum requirement of 10 specimens per group [9].

**Fig. 1:**
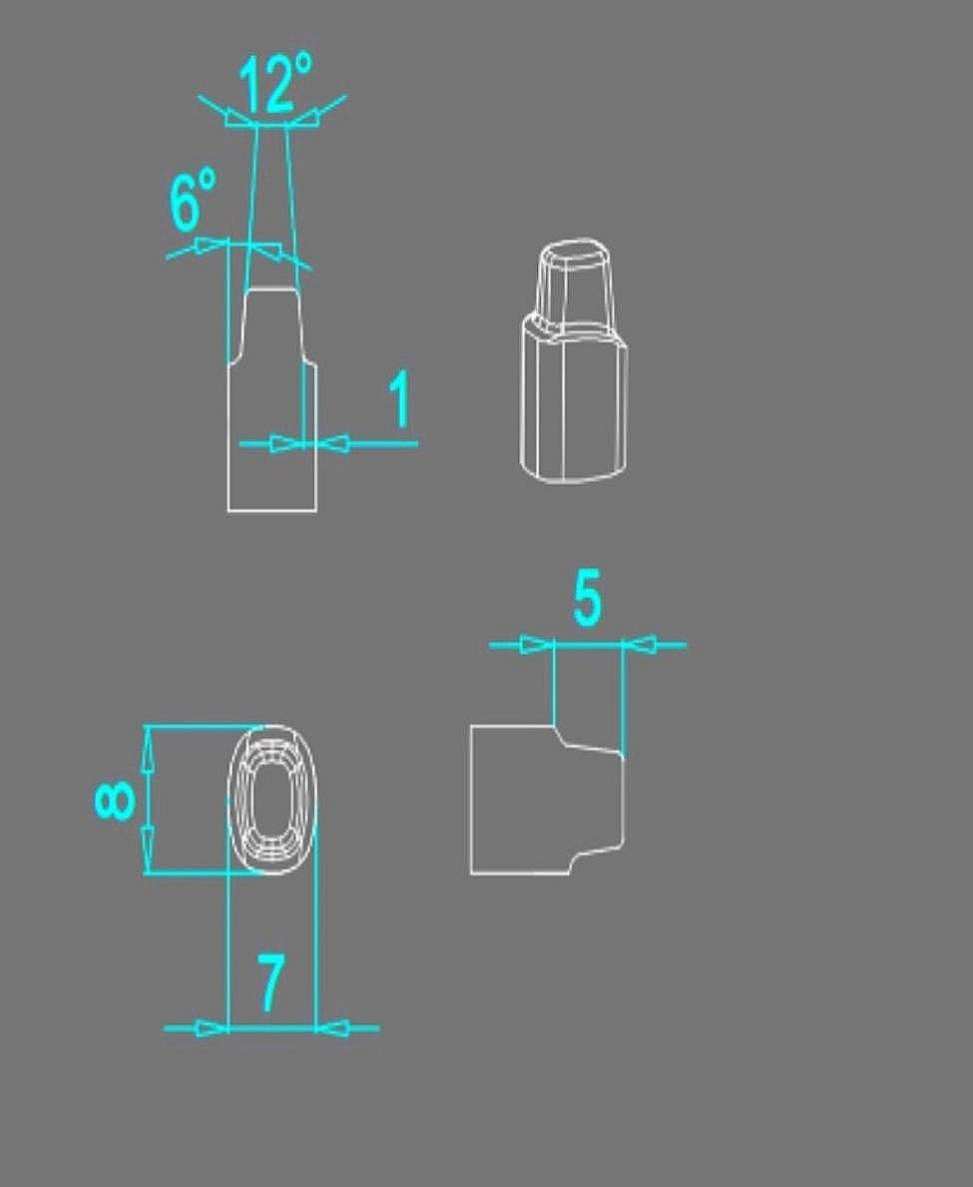
Illustration of the die model

**Fig. 2:**
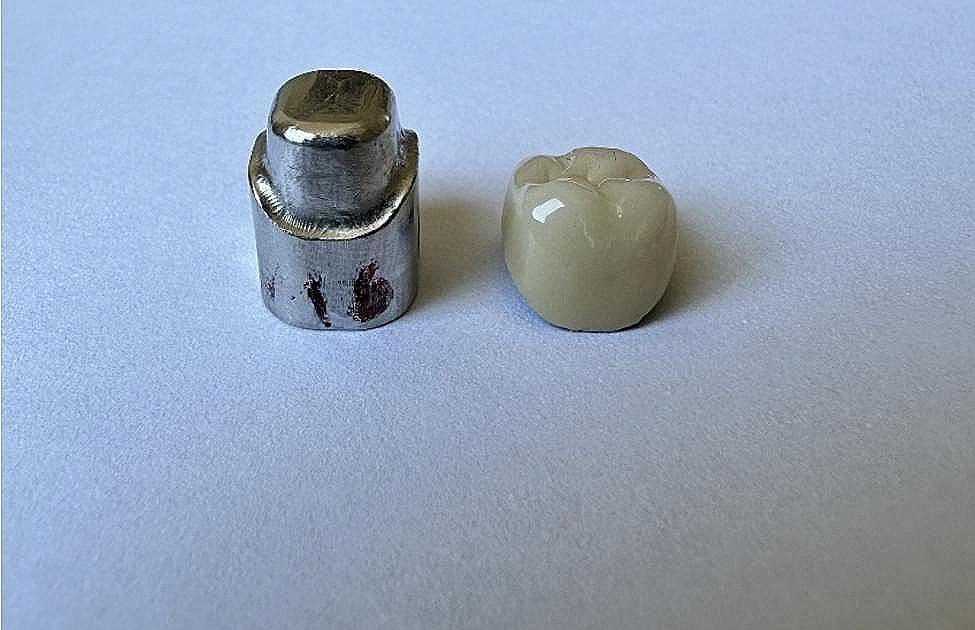
CoCr die model fabricated by CAD/CAM milling

A crown model of 2 mm occlusal reduction and 1 mm of chamfer finish margin, with a total occlusal convergence of 12 degrees representing a prepared maxillary first molar, was designed using Solidworks software (Fig. 1), and this design was used to mill 50 (*n* = 10) identical Co-Cr dies (CC Solar; Camcube) using CAD/CAM technology (M30; Camcube) (Fig. 2).

The die model was scanned with the help of a 3D scanner (Medit T510). The metal copings were designed using CAD software (Exocad GmbH) with a 25-µm cement space. The metal copings were 0.5 mm thick, and the cement gap was set to begin 0.5 mm occlusal from the marginal edge. For use on different devices during production, the data was stored in a standard tessellation language (STL) file. The compositions of metal discs and powders used in coping production are given in Table 1.

**Table 1:** Materials used in the study

Material	Composition	Coefficient of thermal expansion (25–500 °C) Value of Extension	Manufacturer
Kera C; Casting metal	Co 60%, Cr 24.5%, W 8.8%, Nb 2.3%, V 1.8%, Mo 1.2%, Si 0.87%, Fe 0.08%, Al 0.003%, C 0.009%	14,5 × 10^− 6^ K^− 1^	Eısenbacher Dentalwaren ED GmbH
CC Solar ; Hard milling disc	Co 66%, Cr 27%, Mo 6%, Further elements (Si, Mn) Traces	14.3 × 10^− 6^ K^− 1^	Camcube, MESA
Ceramill Sintron; Soft Metal blank	Co 66.0%, Cr 28.0%, Mo 5.0%, Further elements (Mn Si Fe) < 1%, Further elements (C) < 0.1%, Organic binder (for blanks in blank condition) 1–2%	14.5 × 10^–6^ K^− 1^	Hersteller, Amann Girrbach GmbH
EOS Cobalt Chrome SP2; Selective Laser Sintering powder	Co 62–66% Cr: 24–26%, Mo 5 − 7%, W: 4–6%, Si: max. 0.8–1.5%, Mn max. 1.5%, Fe max. 0.7%	13.9–14.3 × 10^− 6^	EOS GmbH - Electro Optical Systems
Mediloy S-Co ; Selective Laser Meltingpowder	Co 63.9%, Cr 24.7%, W 5.4%, Si 1%	14.0/13.7 × 10^− 6^ K^− 1^	BEGO Bremer Goldschlägerei Wilh. Herbst GmbH & Co. KG
Super Porcelain EX3; Noritake; Kuraray (Porcelain construction)	Potassium-aluminosilicate glass, inorganic pigments, glycerol, butane-1,3-diol, etc.	12.3 × 10^− 6^ K^− 1^	Noritake; Kuraray Dental Supply Co, Ltd

Co: Cobalt, Cr: Chromium, W: Tungsten, Nb: Niobium, V: Vanadium, Mo: Molybdenum, Si: Silicon, Fe: Iron, Al: Aluminum, C: Carbon, Mn: Manganese.

Using the STL file, wax blocks (OM Dental) were milled to create wax patterns for conventional casting by the CAM unit (VHF K5). Wax copings were invested for conventional casting. After the elimination of the wax, the Co-Cr alloy (Kera C; ED GmbH) was cast in an electric induction furnace (Protherm Furnaces; Turkey). Airborne particle abrasion using 250 μm aluminum oxide particles (Kuhmichel) under 0.4 MPa pressure was used to remove investment and casting residues.

In the HM group, metal copings were created by milling fully sintered Co-Cr alloy blocks (CC Solar, Camcube) using the previously designed and saved STL file on the 5-axis CAM unit (M30; Camcube). In the SM group, presintered Co-Cr alloy blocks (Ceramill Sintron; Amann Girrbach) were used for milling, and the metal copings were designed to be 11% larger. According to the manufacturer’s instructions, copings were sintered in a sintering furnace (Ceramil Argotherm 2; Amann Girrbach) at 1280 degrees under 1 bar of pressurized argon gas.

In the SLS group, an Eosint M270 (EOS GmbH; Germany) device with an approximately 200 W Yb fiber laser was used to create metal copings using Co-Cr alloy powder (EOS Cobalt Chrome SP2). Stress-relieving of the copings was done under an argon atmosphere up to 750 °C gradually in accordance with the manufacturer’s instructions. In the SLM group, a Mysint 100 Dual Laser (Sisma Spa) device with a 200 W 2x fiber laser was used to create metal copings using Co-Cr alloy powder (Mediloy S-Co; Bego GmbH). Stress-relieving annealing at 650 to 800 °C was conducted gradually according to manufacturer recommendations. All copings were assessed after production and put on the dies for measurement.

**Fig. 3:**
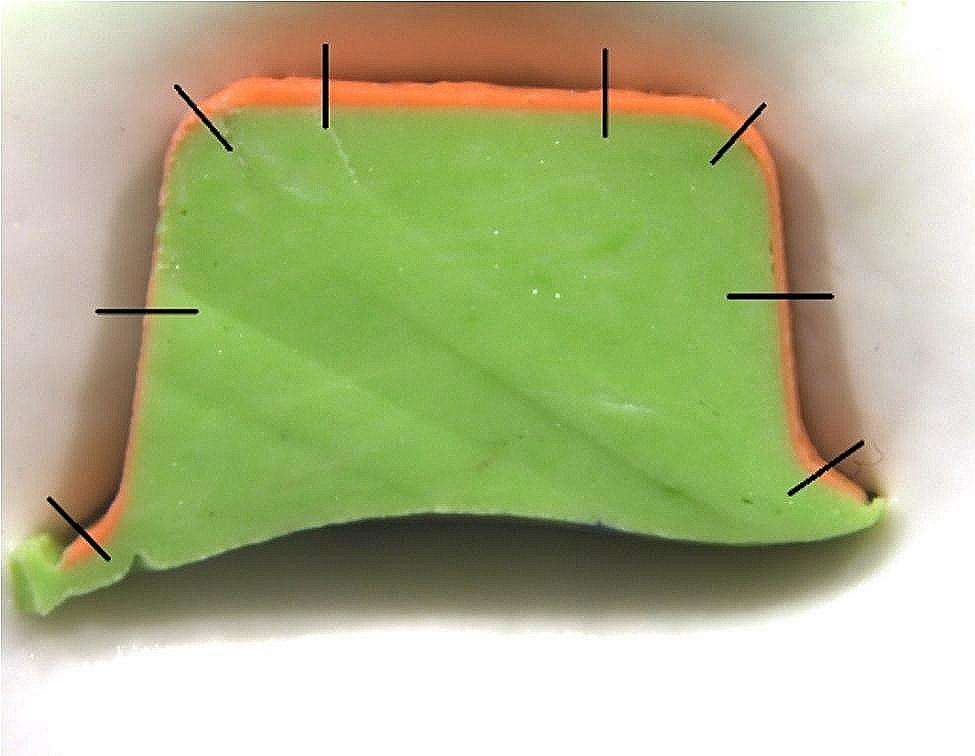
Measurement areas of the cement replica

The silicone replica technique was used to gather measurements. Light-body silicone (Elite HD + Light-Body Fast Set; Zhermack) that had been automixed was applied to the framework in order to simulate the cement space. The coping was placed on the master model and remained for 5 min until the light-body silicone was set up under finger pressure by the same researcher. [5, 18] The coping was taken off, and heavy-body silicone (Elite HD + Putty Soft Fast Set; Zhermack) was poured into the light-body dublicate. The resulting replicate was divided into four portions, mesiodistally and buccolingually. Sections were photographed at x80 magnification using a stereomicroscope (Leica M205 C). The images transferred to the computer were measured by the same researcher using the LAS V4.13 analysis software from the pen-marked spot in the middle of the measurement area. In each image, 1 marginal, 1 axial, 1 axio-occlusal, and 1 occlusal discrepancy measurement was made (Fig. 3). Thus, for each coping, 16 measurements were made for a total of 800 measurements for 50 copings. Mean discrepancy values were measured, and data were recorded.

**Fig. 4:**
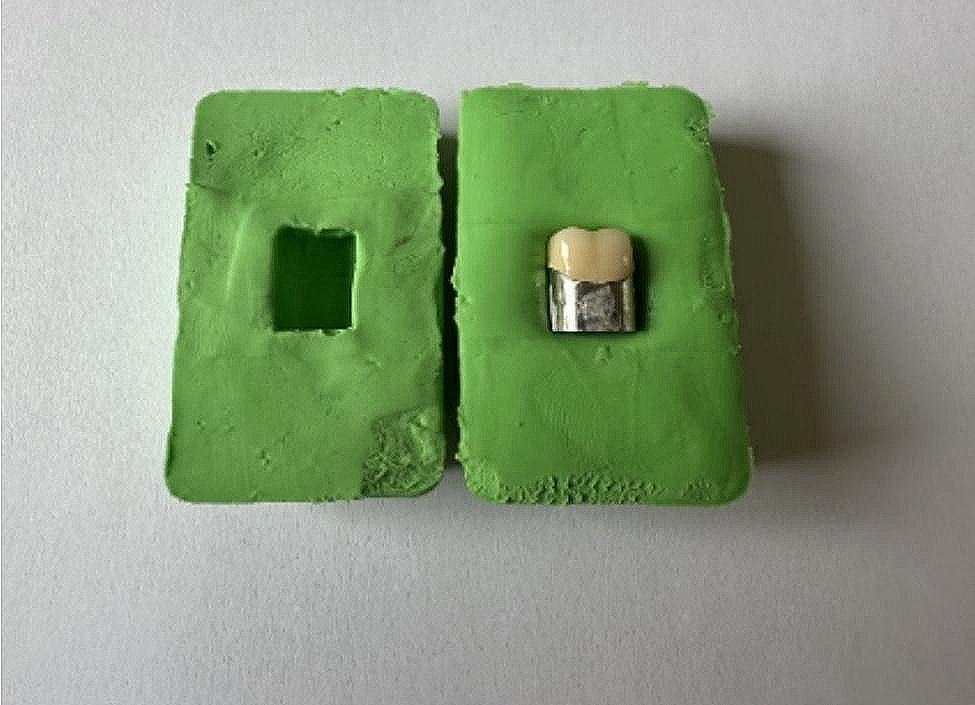
Silicone mold for the fabrication of ceramic veneers

Following these measurements, feldspathic ceramic (Super Porcelain EX3; Noritake; Kuraray) was applied to the copings. Prior to ceramic veneering, the copings underwent airborne-particle abrasion with 250-µm aluminum oxide powder (Kuhmichel) at a pressure of 0.4 MPa. Subsequently, one of the copings underwent ceramic veneering and a silicone mold was created (Fig. 4). The remaining ceramic veneers were then fabricated using a silicone mold for standardization using the same workflow. After degassing (oxidation) the metal frameworks up to 980 °C, Universal Paste Opaque was applied to the copings and fired under 96 kPa vacuum with a heat rate of 55 °C/min, raising from 600 °C up to 960 °C in the furnace (Dekema Austromat, Dental-Keramiköfen GmbH). Cervical, body, enamel, and translucent firings were conducted, respectively, in the furnace (Vacumat 500, Vita Zahnfabrik) at 96 kPa vacuum level, raising the temperature by 45 °C/min heat rate from 600 °C to 930 °C. Glaze firing was conducted in the furnace (Vacumat 300, Vita Zahnfabrik) with a heat rate of 50 °C/min, raising from 650 °C to 910 °C without a vacuum. Porcelain firing was followed by obtaining the second measurement. Similar to the metal copings, the discrepancy of the metal-porcelain crown was measured.

### Statistical analysis

The data were analyzed using the trim method with the WRS2 package in the R program. Compliance with the normal distribution was analyzed with skewness-kurtosis coefficients (± 3). The ROBUST three-way analysis of variance (ANOVA) method was used to compare the discrepancy values that do not comply with normal distribution according to method, area, and porcelain firing. The analysis results are presented as a trimmed mean ± standard error. The significance level was taken as *P* < .050.

## Results

The main effect of the fabrication method, irrespective of the measurement area and porcelain firing, was found to be statistically significant on the discrepancy values (*P* < .001) (Table 2). The mean value of the discrepancy was obtained as 90.1 μm in the HM method, 74.9 μm in the SLM method, 67.7 μm in the SM method, 61.6 μm in the SLS method, and 63 μm in the C method (Table 3.). The values obtained from the SM, SLS, and C methods were similar. The mean values of the SLM and SM methods were similar. The mean value of the discrepancy obtained in the HM method differed from all other methods (*P* < .001) (Table 3.).

**Table 2:** Q: Three-way ROBUST ANOVA test results

	Q	*p*
Method	289	< 0.001
Area	530	< 0.001
Porcelain Firing	3548	0.001
Method*Area	268	0.001
Method* Porcelain Firing	320	0.001
Area * Porcelain Firing	133	0.001
Method* Area * Porcelain Firing	223	0.001

**Table 3:** Mean discrepancy values and standard deviations across different fabrication methods (µm)

Porcelain Firing	Methods				
	HM	SLM	SM	SLS	C
Before	152.1 ± 5.49^A^	120.6 ± 3.08^C^	100.3 ± 2.81^D^	89.3 ± 2.42^E^	94 ± 3.17^DE^
After	34.9 ± 1.49^B^	34 ± 0.98^B^	37.6 ± 1.62^B^	35.4 ± 1.51^B^	35.8 ± 1.17^B^
Total	90.1 ± 4.49^a^	74.9 ± 3.07^b^	67.7 ± 2.45^bc^	61.6 ± 2.15^c^	63 ± 2.28^c^

Regardless of all variables, if the production methods were compared solely based on the results after porcelain firing, and the primary effect of the production techniques was ignored, there was no difference observed in any method.

The main effect of measurement areas on the discrepancy values, regardless of manufacturing methods and porcelain firing, was determined to be statistically significant (*P* < .001) (Table 2). The mean discrepancy values obtained were as follows: 74.1 μm in the marginal area, 47.7 μm in the axial area, 74.4 μm in the axio-occlusal area, and 87.1 μm in the occlusal area (Table 4). The lowest mean discrepancy value was observed in the axial area, whereas the highest was in the occlusal area (*P* < .001). While the mean discrepancy value in the marginal area was similar to that in the axio-occlusal part, values in the other regions exhibited variations from each other (Table 4).

The main effect of porcelain firing, regardless of manufacturing methods and measurement area, was statistically significant on the discrepancy values *(P* = .001) (Table 2.). The mean value before the porcelain firing was 109.2 μm, obtained at 35.3 μm after firing (Table 4).

**Table 4:** Mean discrepancy values and standard deviations across measurement areas (µm)

Porcelain Firing	Area				
	Marginal	Axial	Axio-occlusal	Occlusal	Total
Before	107.4 ± 2^A^	78.7 ± 2.32^C^	120.9 ± 3.38^E^	135.9 ± 4.58^G^	109.2 ± 1.70
After	43.2 ± 0.76^B^	20.6 ± 0.74^D^	33.1 ± 0.77^F^	45.1 ± 1.41^B^	35.3 ± 0.58
Total	74.1 ± 2.01^a^	47.7 ± 1.93^b^	74.4 ± 2.92^a^	87.1 ± 3.38c	70 ± 1.32

There was a statistically significant interaction between the fabrication method and the measurement area in relation to the discrepancy values (*P* = .001) (Table 2). The highest discrepancy was obtained in the occlusal area in the HM method, with a mean value of 135.1 μm. The mean discrepancy was similarly high in the axio-occlusal area in the HM method with 106.5 μm, in the axio-occlusal area in the SLM method with 87.8 μm, and in the occlusal area in the SLM method with 88.3 μm. The values in other interactions differed from those obtained in the occlusal area of the HM method. The lowest value obtained was in the axial area of the SM method with 39.2 μm (Table 5).

**Table 5:** Descriptive statistics and multiple comparison results of discrepancy values (µm)

Area	Porcelain Firing	Methods				
		HM	SLM	SM	SLS	C
Marginal	Before	128.4 ± 4.78^ABC^	100 ± 3.46^RST^	107.8 ± 4.58^CRS^	93 ± 3.46^İST^	108.8 ± 4.06^CRS^
	After	41.8 ± 2.21^DEFGH^	39.1 ± 1.1^DEFG^	46.3 ± 1.56^EHQ^	47 ± 1.78^EHQ^	42.4 ± 1.63^EGH^
	Total	85.3 ± 5.9^ABC^	68.3 ± 4.11^BCGHİ^	75.3 ± 4.46^ABCG^	69.1 ± 3.24^BCGH^	74.6 ± 4.64^ABCG^
Axial	Before	73.2 ± 5.32^İJK^	95.1 ± 6.35^İRST^	64.8 ± 4.5^JKQ^	81.2 ± 4.63^İKT^	79.6 ± 3.81^İK^
	After	21.3 ± 1.72^LM^	20.1 ± 1.69^L^	16.7 ± 1.43^L^	20.5 ± 1.58^L^	24.4 ± 1.82^LMO^
	Total	45.3 ± 4.18^DE^	55.6 ± 5.66^DEGHİ^	39.2 ± 3.59^D^	49.2 ± 4.31^DEİ^	51 ± 3.97^DEHİ^
Axio-occlusal	Before	179.6 ± 3.82^N^	143.7 ± 3.78^A^	106.7 ± 4.14^CRS^	86.8 ± 4.56^İKST^	88.1 ± 6.16^İKST^
	After	34.7 ± 2.44^DFGO^	34.2 ± 1.28^DF^	33.6 ± 1.21^F^	31.8 ± 2.14^DFMO^	32.4 ± 1.86^DFO^
	Total	106.5 ± 9.21^AF^	87.8 ± 7.04^ABCFG^	68.4 ± 4.98^ABCEGHİ^	58.5 ± 4.19^DEGHİ^	58.2 ± 4.73^CDEGHİ^
Occlusal	Before	227.8 ± 6.53^P^	139.1 ± 4.83^AB^	119.8 ± 4.73^BCR^	96.5 ± 6.35^İRST^	104.4 ± 9.64^ABCİKRST^
	After	44.6 ± 4.92^DEFGHOQ^	41.4 ± 1.41^DEGH^	56.1 ± 3.45^HJQ^	43.4 ± 3.9^DEFGHQ^	44.1 ± 2.37^DEGH^
	Total	135.1 ± 11.91^F^	88.3 ± 6.55^ABF^	86.8 ± 4.81^AB^	68.8 ± 4.89^ABCGHİ^	70.6 ± 5.93^ABCEGHİ^

(Trimmed mean ± Standard Error; ^a−c^: There is no difference between main effects with the same letter; ^A−T^: There is no difference between interactions with the same letter. HM: Hard metal milling, SLM: Selective laser melting, SM: Soft metal milling, SLS: Selective laser sintering, C: Casting)

The interaction between the fabrication method and porcelain firing was found to be statistically significant for the discrepancy values, irrespective of the measurement area (*P* = .001) (Table 2). The highest discrepancy, with a mean value of 152.1 μm, was obtained before porcelain firing in the HM method and differs from all other metal production methods. SLS and C methods were similar before porcelain firing. The highest mean discrepancy value after porcelain firing was 37.6 μm in the SM method, though the mean discrepancy value was statistically similar and decreased in all methods. The SLM method received the lowest mean value of 34 μm after porcelain firing (Table 3).

The interaction of measurement area and porcelain firing was statistically significant on the discrepancy values (*P* = .001) (Table 2). The highest mean value of 135.9 μm was obtained in the occlusal area before porcelain firing and differed from all other interactions. After porcelain firing, mean discrepancy values decreased in all areas. The lowest mean value, 20.6 μm, was obtained in the axial area after porcelain firing and differed from all other interactions (Table 4).

The interaction of method, area, and porcelain firing was statistically significant on the discrepancy values (*P* = .001) (Table 2). The highest discrepancy was obtained in the HM method in the occlusal area before firing, with a mean value of 227.8 μm (Table 5). The lowest mean value of discrepancy, 16.7 μm, was obtained in the SM method in the axial area after porcelain firing (Table 5).

## Discussion

This in vitro study aims to assess how metal fabrication techniques affected metal-porcelain crown accuracy before and after the porcelain firing. Findings demonstrated that the methods used to manufacture metal frameworks varied significantly. The mean discrepancy also varied among measurement areas and differed before and after porcelain firing. Consequently, the entire null hypothesis was rejected.

In the methodological aspect, some of the research has shown that finish-line arrangement affects restoration fitting accuracy [26, 33, 38, 39]. A systematic review and meta-analysis [40] revealed that the marginal adaptation with the chamfer finish line preparation was better achieved by soft milling, direct metal laser sintering, and hard milling methods, respectively. In this investigation, the chamfer finish line was preferred for standardization and better adaptation. This could be the reason why the HM group discrepancy value was greater than that of the similar SM and SLS groups.

To evaluate the accuracy of dental restorations, instead of methods such as microCT, which require technical precision [9, 16], the direct-view technique with a microscope [1], and the cross-sectioning technique, which can destroy the cemented copings by cutting [5, 11], a silicone replica technique has been used, which is non-destructive, highly reliable, and sensitive. The silicone replica technique used in the study can also be used for in vivo evaluations, as it allows the evaluation of both marginal and internal discrepancies. Also, the technique is cheap and does not require advanced technology [9, 18, 24, 26, 36]. However, the method is prone to errors caused by human mistakes [7], and it was still difficult to remove the elastomeric film from the crown without tearing.

The same researcher applied finger pressure during the investigation for standardization, despite the fact that the seating force applied to the crown lined with cement cannot be standardized under in vivo conditions. However, it is claimed that variations in sitting force have little effect on the silicone layer thickness [36, 41].

Although the silicone replica technique is considered a reliable and valid method in the literature [5, 24], it has the potential to produce silicone layers of varying thicknesses, which can influence the data. Consequently, current methods such as 3D scanning, which evaluates the cement gap, may be considered for future studies.

The literature accepts that marginal discrepancy values beyond 120 μm are not suitable for restorations [4, 42]. Prior to porcelain firing, only HM had a discrepancy value (152,1 μm) greater than this threshold; however, after porcelain firing, all methods had discrepancy values less than 120 μm. Among the production methods, the highest average discrepancy value after porcelain firing was observed in SM at 37.6 μm, and all methods were statistically similar. Thus, it was found that every fabrication technique demonstrated clinical success in terms of adaptation.

Compared to the soft metal milling method and the other groups, the metal copings produced through the hard metal milling method exhibited significantly higher marginal discrepancy values. This finding aligns with studies conducted by Park et al. [25] and Kim et al. [12]. In contrast, some studies [2, 4, 27] reported that hard milling discrepancy values were lower than those for soft milling. This variance can be attributed to the complex sintering process of the SM group. Pre-sintered soft metal frames reach their maximum density after the milling process is complete, causing the metal framework to contract by approximately 10–11% during the sintering process, ultimately determining its final hardness and dimensions [24–26]. Compared to hard metals, pre-sintered soft metals may exhibit less variation in precision due to their contraction process [4, 12, 15, 25, 27].

The laser sintering group exhibited statistical similarity to the soft metal milling group and displayed lower discrepancy values than the hard metal milling group. The cobalt-chromium powder was precisely created and solidified in small sections, which is responsible for the improved compatibility of metal copings that were laser-sintered [7]. This minimizes the dimensional changes of the alloy, and a more homogeneous, almost completely dense material can be produced [11, 19]. Also, the CAM device vibration during machining, overheating during milling, bur size [2], and bur wear could all have had an impact on the insufficient rounding of the metal blocks, which may have led to the HM group’s discrepancy values. In contrast, additive manufacturing processes, including laser melting (SLM) and laser sintering (SLS) methods, do not allow for bur compensation, distinguishing them from subtractive processes [2, 10, 23, 37].

Although the equipment used in the SLM and SLS methods is very similar, the SLS method had better accuracy than the SLM method in the present study. SLM uses a much higher energy density and a high-power laser that allows the powders to melt completely. SLM has the capability to produce metals with a complete density of approximately 99.8% ^21^, whereas SLS typically yields metals with a lower density [29, 30, 34]. Insufficient energy density during powder irradiation prevents complete melting, leading to a lack of fusion and the emergence of defects [11]. The type of production method can also affect the residual stress level. There are large residual stresses resulting from temperature changes created by laser-based production methods, which are more evident in the SLM group than in the SLS group [22].

Compared to the EOSINT M 270 (LS), the laser spot diameter in the MYSINT 100 (SLM) is substantially lower. The product’s top layer melts when a laser focused on a smaller area in SLM generates additional heat. As the temperature variations between layers increase, the product deforms [29, 30]. Furthermore, there may be differences between these two methods depending on the scanning distance, layer thickness, laser power, laser spot diameter, and scanning speed [4, 30].

In the present study, the conventional casting method showed the best adaptation with the lowest discrepancy values, similar to the study of Park et al. [35] and also less than the HM group, similar to the study of Örtorp et al. [10] It proved that it is still valid despite being a complex and delicate technique that requires more steps than others and has disadvantages such as deformation of the wax model, hardening expansion of the investment, and dimensional change of the molten metal [18, 19, 26]. The discrepancy of the C method was also similar to that of the SLS and SM methods. After conducting a subgroup meta-analysis of gaps examined under a stereomicroscope, Yang et al. revealed that the mean absolute marginal and occlusal gaps of single metal copings made by selective laser sintering were similar to those of copings made by conventional casting [43].

The measurement area with the lowest discrepancy was also along the axial walls before and after porcelain firing, which was similar for all production methods. Örtorp et al. [10] found the lowest discrepancy in the axial wall, which is in line with our findings. Nesse et al. [1] stated that placement along a nonideal path could affect the amount of cement gap along the axial walls. However, these factors were the same for all specimens in the study. In this study, the occlusal and marginal discrepancies were significantly greater than the axial discrepancies, which is consistent with the study of Vojdani et al. [2]. Tamaç et al. [36] and Örtorp et al. [10] also found the highest discrepancy in the occlusal area in their studies. As Hassan and Goo [44] mentioned in their study, one possible explanation for the less discrepancy in axial area is that in narrow areas of the crown, the light body silicon increased intracoronal hydraulic pressure, which could result in greater resistance forces during mimicing the cementation, potentially hindering the crown from seating fully. So that relatively flat areas such as occlusal area may act as a pool for cement or impression material. The relatively low discrepancy observed in the marginal area may have been caused by creating the cement gap from 0.5 mm above.

In comparison with other CAD/CAM methods, the higher values observed in the hard milling group in all areas, particularly in the occlusal area before porcelain firing, are believed to be due to the absence of post-heat treatment, which is already present in the workflow of all other fabrication methods.

Previous studies reported that discrepancies increased after ceramic firing [11, 22, 23]. When high temperatures are applied to metal copings during firing cycles, this can lead to dimensional changes or distortions that eventually lower the marginal fit of metal-ceramic restorations [11, 16, 26]. However, this structural deformation is caused by the type of alloy, the shrinkage of the ceramic during porcelain firing, and the difference in the thermal expansion coefficient between the metal and the ceramic [16, 18, 24, 26]. Additional factors that contribute are the creep of the alloys at high temperatures and residual stress on the frameworks from the stages prior to firing [16]. Nevertheless, a number of investigations found no appreciable variations in marginal discrepancy following ceramic applications [11, 13, 16, 18, 24, 45].

In the present study, discrepancies decreased after porcelain firing in all groups. It was thought that during the ceramic firing, the volume percentage of the major faults had decreased. Also, Hong et al. [11] assessed the marginal fit of metal-ceramic crowns fabricated by casting and two SLM processes. According to their findings, the SLM group with small porosity revealed a decrease in marginal discrepancy values after porcelain firing, while the others increased. This was attributed to porcelain firing, which acted as a secondary heat treatment similar to annealing, ultimately altering the alloy’s metallurgical structure [16, 37] and resulting in changes in the dimensions and marginal fit of metal-ceramic crowns. [11, 46].

The methodological variations among studies such as differences in the number of measurements performed, the use of distinct materials, the sensitivity of CAD-CAM systems and software, dimensional stability of the impression material, and the measurement method used can all have an impact on the accuracy of restoration fittings [9, 14, 18, 26]. In vitro design of the study and controlled laboratory environment constitute a drawback. Additional in vivo investigations are required to compare the fit of metal-ceramic restorations.

## Conclusions

The following conclusions were made in light of the in vitro study’s findings:

1. All fabrication methods had acceptable values in terms of metal-porcelain crown fit (< 120 μm).
2. Among the different fabrication methods’s main effects, the HM method exhibited the highest discrepancy, while the SLS method, which was statistically similar to the SM method, showed the lowest discrepancy.
3. Porcelain firing did not increase the discrepancy but significantly reduced it. Consequently, all methods showed similar results after porcelain firing.
4. The highest discrepancy was observed on the occlusal area, and the lowest discrepancy was observed on the axial area of the coping.

Even though high technology allows additive and subtractive methods to save time, they still need to be developed to be an alternative to conventional casting methods.

## Data Availability

The data sets used and/or analysed during the current study are available from the corresponding author upon reasonable request.

## Abbreviations

CAD/CAM: Computer aided design/ Computar aided manufacturing
C: Casting
HM: Hard metal milling
SM: Soft metal milling
SLM: Selective laser melting
SLS: Selective laser sintering
